# Outbreak of Tuberculosis Associated with a Newly Identified *Mycobacterium tuberculosis* Genotype — New York City, 2010–2013

**Published:** 2013-11-15

**Authors:** Jillian Knorr, Jeanne Sullivan Meissner, Bianca R. Perri, Shama Desai Ahuja

**Affiliations:** New York City Dept of Health and Mental Hygiene

In January 2010, the New York City (NYC) Department of Health and Mental Hygiene (DOHMH) identified a tuberculosis (TB) case caused by *Mycobacterium tuberculosis* with a genotype not reported previously in the United States (1). The patient was evaluated for TB while incarcerated but was released before the diagnosis was confirmed and before beginning TB treatment. The patient, who had a history of homelessness and clinical characteristics suggesting infectiousness, could not be located by DOHMH for 13 months. Numerous efforts were made to locate the patient, including queries to shelters, jails, and infection-control staff members at local hospitals. The patient was located after he had an abnormal chest radiograph result following referral by a local jail to a hospital emergency department (ED) for symptoms of alcohol withdrawal; he died from complications of liver cirrhosis 5 days later, without having started TB treatment. During February 2012–May 2013, DOHMH identified four additional patients with the same TB genotype. All five patients were U.S.-born black men aged 52–57 years. Four had a history of substance abuse; three had a history of homelessness; and two had a history of incarceration. All patients had drug-susceptible TB and were negative for human immunodeficiency virus. Three patients completed TB treatment. One patient, who was homeless at the time of diagnosis, began TB treatment but was lost to follow-up by DOHMH.

Contact investigation was conducted per routine NYC protocol (2) and included contact elicitation at one jail, two homeless shelters, two health-care facilities, and one drug treatment facility. During the outbreak investigation, epidemiologists reinterviewed all patients except the index patient. Among three patients with a history of homelessness, all reported spending time living on the street. Although no patient named another patient as a contact, four patients spent considerable time near the same NYC transportation hub. Three patients, including the index patient, had multiple visits to the same NYC hospital ED for care related to alcohol withdrawal and other health issues in the years around their TB diagnoses. The index patient made several visits to this ED during the 13 months when he could not be located by DOHMH. Although it is not possible to definitively determine where transmission occurred, multiple epidemiologic links among patients indicate recent transmission of a new TB strain in NYC.

Genotyping combined with epidemiologic expertise enabled DOHMH to detect an outbreak among persons not previously known to be linked and to identify possible sites of TB transmission that were not apparent from contact investigation alone. DOHMH also identified a social network of homeless persons who primarily lived on the street and had a history of substance abuse and frequent ED use. DOHMH and the NYC Department of Homeless Services (DHS) have a history of working collaboratively to detect and treat TB among homeless persons residing in shelters. However, TB control among homeless persons living on the street presents unique challenges. In conjunction with this investigation, DOHMH is working with DHS, local hospitals, and other organizations to improve capacity for locating TB patients lost to DOHMH supervision and to identify mechanisms for enhancing TB diagnosis, treatment, and case management for homeless persons who live on the street.

Although the burden of TB in the United States has largely shifted from U.S.-born to foreign-born populations over the past 2 decades (3), this outbreak is a reminder that transmission continues to occur among U.S.-born persons and highlights the need for TB controllers, ED health-care providers, and others to remain vigilant for TB among persons with a history of homelessness, substance abuse, or other TB risk factors (4). Although previous outbreaks have been linked to homeless shelters (5–7), this investigation revealed other sites of possible transmission, including a hospital ED and a public transportation hub. DOHMH continues to monitor TB genotyping results to identify additional patients in this outbreak.
